# Thymic Stromal Lymphopoietin Induction Suppresses Lung Cancer Development

**DOI:** 10.3390/cancers14092173

**Published:** 2022-04-27

**Authors:** Ranya Guennoun, Jennet Hojanazarova, Kathryn E. Trerice, Marjan Azin, Matthew T. McGoldrick, Erik B. Schiferle, Michael P. Stover, Shadmehr Demehri

**Affiliations:** 1 Center for Cancer Immunology, Center for Cancer Research, Massachusetts General Hospital and Harvard Medical School, Boston, MA 02114, USA; g.ranya@wustl.edu (R.G.); jhojanazarova@mgh.harvard.edu (J.H.); ktrerice@mgh.harvard.edu (K.E.T.); mazin@mgh.harvard.edu (M.A.); mmcgold3@jhmi.edu (M.T.M.); ebs2154@bu.edu (E.B.S.); micsto@med.umich.edu (M.P.S.); 2 Cutaneous Biology Research Center, Department of Dermatology, Massachusetts General Hospital and Harvard Medical School, Boston, MA 02114, USA

**Keywords:** thymic stromal lymphopoietin, lung cancer, Kras, CD4^+^ T cell, antitumor immunity

## Abstract

**Simple Summary:**

The recurrence rate for lung cancer is high after the removal of the primary tumor. Herein, we demonstrate the potential of immunotherapy against lung cancer by examining the impact of Thymic Stromal Lymphopoietin (TSLP) cytokine induction on early lung cancer development. TSLP induction suppresses the development of invasive lung tumors in a mouse model of spontaneous lung cancer. This cancer suppression is dependent on CD4^+^ T cells, which highlights the role of adaptive immune response in protection against lung cancer progression.

**Abstract:**

Lung cancer is the leading cause of cancer deaths in the United States and across the world. Immunotherapies, which activate tumor-infiltrating cytotoxic T lymphocytes, have demonstrated efficacy for the treatment of advanced-stage lung cancer. However, the potential for harnessing the immune system against the early stages of lung carcinogenesis to prevent cancer development and recurrence remains unexplored. Using a mouse model of lung adenocarcinoma, we investigated the effects of thymic stromal lymphopoietin (TSLP) induction on early cancer development in the lungs. Herein, we demonstrate that systemic TSLP induction suppressed spontaneous lung cancer development in Kras^G12D^ mice. TSLP drove a significant CD4^+^ T cell response to block lung cancer progression from atypical alveolar hyperplasia to adenocarcinoma. Our findings suggest that TSLP can be used in the early stages of lung cancer development to trigger a lasting immunity in the tissue and prevent the development of advanced disease.

## 1. Introduction

Lung cancer is the primary cause of cancer-related deaths in the US and the world [1,2]. Non-Small Cell Lung Cancer (NSCLC) comprises 85% of lung cancer diagnoses, and 40% of those are adenocarcinomas, a glandular neoplasm [3]. Surgery for the early and locally advanced disease remains the main treatment option for patients diagnosed with NSCLC. However, despite curative resection, recurrence rates remain high with 30–75% of NSCLC patients developing lung cancer after surgical removal of their first tumor, and with up to 80% developing recurrence within 2 years after diagnosis [4]. Thus, novel strategies to block lung cancer development and recurrence are urgently needed.

Among the potential therapeutic approaches for early lung cancer, current immunotherapies have several limitations. Apart from immune-related adverse events [5], immunotherapies that depend on high tumor-infiltrating lymphocytes at baseline may have minimal efficacy against early malignancies, which are largely non-immunogenic “cold” tumors [6]. Early malignant lesions in the lungs have a low mutational burden, and a less inflamed microenvironment, with low infiltration of CD8^+^ T cells, which may lead to poor responses to checkpoint blockade therapy, neoantigen-based vaccines and engineered T cells [7,8]. Thus, identifying novel immune inductive pathways that can target tumors at the early stages of their development is imperative to generate effective therapeutic and preventative approaches against poorly inflamed early malignant lesions. In addition to suppressing cancer development, such strategies used in the neoadjuvant and adjuvant settings will create an opportunity for a lasting antitumor immunity that prevents cancer recurrence.

We have previously demonstrated that the induction of Thymic Stromal Lymphopoietin (TSLP) leads to a robust antitumor CD4^+^ T helper 2 (Th2) cell immunity that suppresses skin and breast cancer development [9,10]. TSLP is an epithelium-derived alarmin cytokine that is a master regulator of allergic inflammation at barrier organs like the skin and lungs [11]. Interestingly, epidemiological data suggest an inverse relationship between allergic disease and lung cancer risk [12,13], which could be explained by higher serum TSLP levels in the patients. Accordingly, we hypothesize that TSLP induction in patients with early lung adenocarcinoma may prevent cancer progression and recurrence. TSLP can promote differentiation of CD4^+^ T cells in the Th2 cells either directly by binding to TSLP receptor (TSLPR) expressed on CD4^+^ T cells or through activation of dendritic cell and other antigen-presenting cells, which can then polarize CD4^+^ T cells to differentiate into the Th2 phenotype [14]. TSLP-activated CD4^+^ Th2 cells create an immune milieu rich in IL-4, IL-5, IL-13 as well as TNF-alpha [15]. Although baseline TSLP expression in the tumor microenvironment has been associated with tumor promotion [16,17,18,19,20], harnessing the potential of TSLP induction to trigger a strong CD4^+^ T cell response, which has been previously shown to be cancer suppressive [9,10,21], may provide a powerful therapeutic approach against lung cancer.

To determine the effects of TSLP induction on early carcinogenesis in the lung, we crossed a mouse model of spontaneous lung adenocarcinoma, Kras^+/G12D^ (Kras^G12D^), with K14-TSLP^tg^ (Tslp^tg^) mice. Tslp^tg^ Kras^G12D^ mice developed a significantly lower lung tumor burden compared with Kras^G12D^ mice. Tslp^tg^ Kras^G12D^ lung tumors were mainly composed of lower-grade atypical alveolar hyperplasia and adenoma compared with adenocarcinoma in Kras^G12D^ lung. We found increased numbers of lymphoid aggregates in Tslp^tg^ Kras^G12D^ lungs and increased T cell infiltration in Tslp^tg^ Kras^G12D^ lung tumors compared with Kras^G12D^ lungs and lung tumors, respectively. Finally, CD4^+^ T cell depletion reversed the protective impact of TSLP against lung carcinogenesis in TSLP overexpressing mice. Our results indicate that TSLP induction can generate a strong, long-lasting cancer suppressive immune response in the lung and provide a potential therapeutic approach for the prevention and treatment of lung cancer.

## 2. Materials and Methods

### 2.1. Mice

All mice were housed under pathogen-free conditions in an animal facility at Massachusetts General Hospital in accordance with all animal care and relevant ethical regulations. Kras^+/G12D^ (B6.129S-Krastm3Tyj/Nci or K-rasLA2) mice were obtained from NCI Mouse Repository. K14-TSLP^tg^ mouse strain was a gift from Dr. Andrew Farr (University of Washington, Seattle, WA, USA).

### 2.2. Statistical Analysis

A two-tailed Mann-Whitney *U* test was used as the test of significance for tumor counts, T cell counts and other quantitative measurements. A two-tailed Fisher’s exact test was used to compare tumor grade distributions. For multiple group comparisons, the Kruskal-Wallis test with Dunn’s multiple comparison post-hoc test was used. A *p* value < 0.05 was considered significant. All bar graphs show mean + SD. 

### 2.3. Study Approval

Animal studies were approved by Massachusetts General Hospital Institutional Animal Care and Use Committee (IACUC). 

### 2.4. Spontaneous Lung Cancer Studies

All mice were harvested at postnatal day 100. Lungs and other tissues were collected for histological analysis.

### 2.5. Histology and Immunofluorescence 

Mouse lung was fixed in 4% paraformaldehyde (Sigma-Aldrich, Darmstadt, Germany; P6148) and incubated at 4 °C overnight on a shaker. The next day, tissues were washed in 1x PBS and dehydrated in ethanol. Next, lungs were processed, embedded in paraffin, and cut into 5 µm sections. Then lungs were deparaffinized and stained with Hematoxylin (Sigma Aldrich, St. Louis, MO, USA; GHS132) and Eosin (Leica Biosystems; Buffalo Grove, IL, USA; 3801619). For Immunofluorescence assays, tissue slides were deparaffinized and rehydrated. Then tissues were permeated with 1x PBS supplemented with 0.2% *v*/*v* Triton X-100 (Thermo Fisher Scientific, Waltham, MA, USA; BP151). For all washing steps, slides were dipped in three, three-minute rounds of 1x PBS supplemented with 0.1% *v*/*v* Tween 20 (Sigma-Aldrich; P1379). Antigen retrieval was performed in antigen unmasking solution (Vector Laboratories, Burlingame, CA, USA; H-3300) using a Cuisinart pressure cooker for 20 min at high pressure. Slides were then washed, and incubated in a blocking buffer composed of 5% *v*/*v* goat serum (Sigma-Aldrich; G9023), and 5% m/v bovine serum albumin (Thermo Fisher Scientific; BP1600) for 1 h at room temperature (RT). Then slides were incubated overnight at 4 °C with primary antibodies (Table S1). The next day, slides were washed and incubated at RT with fluorochrome-conjugated secondary antibody diluted in blocking buffer for 2 h. After washing, slides were counterstained with 1:4000 DAPI (Thermo Fisher Scientific; D3571) at RT for 5 min. Then slides were washed and mounted with Prolong Gold Antifade Reagent (Thermo Fisher Scientific; P36930).

For imaging, slides were scanned using Nanozoomer s60 digital scanner (Hamamatsu Corp. Bridgewater, NJ, USA) or Axio Scanner (Axio Scan.Z1, Zeiss, Jena, Germany). H&E images were analyzed using NDP.view 2+ software or the Zeiss ZEN Image processing software. Quantification of cell population in tumor and lymphoid aggregates were performed manually using Cell Counter on NDP.view 2+ or with HALO Digital Pathology Software for Image Analysis (HPF, Indica Labs, Albuquerque, NM, USA). Positive cells were quantified within 20× frame by comparing fluorescent intensity of double- (DAPI^+^ CD3^+^/Ki67^+^) and/or triple-(DAPI^+^ CD3^+^ CD4^+^) positive cells to the background (Table S1).

### 2.6. CD4^+^ T Cell Depletion

After weaning (postnatal day 30), Tslp^tg^ Kras^G12D^ and Kras^G12D^ mice were injected intraperitoneally with 750 µg in 200 µL of sterile PBS with anti-CD4 (αCD4, Table S1) or IgG (control; Sigma-Aldrich, I4131) antibody for the first dose. One week later, the mice were then injected with 250 µg of αCD4 or IgG antibody in 200 µL of sterile PBS weekly until postnatal day 100. To determine whether CD4^+^ T cells were systemically depleted, we performed flow cytometry on mice spleen, lymph node and blood at the time of harvest.

### 2.7. Flow Cytometry

Lymph nodes were digested in 0.4% *v*/*v* collagenase IV (Worthington Biochemical, Lakewood, NJ, USA; LS004188) in RPMI 1640 (Thermo Fischer Scientific; 11-875-093) and incubated at 37 °C shaker for 30 min. Then lymph nodes and spleen were homogenized by passing the tissue through 70 µm filters and washed with R10 [500 mL of RPMI 1640, 50 mL of fetal bovine serum (Sigma-Aldrich; F0926), 5 mL of Pen Strep Glut (Thermo Fischer Scientific; 10378016), and 0.5 mL of 2-Mercaptoethanol (Fischer Scientific; 21-985-023)] and centrifuged at 1200 rpm for 7 min. The spleen and blood samples were lysed in 1x RBC lysis solution (BioLegend, San Diego, CA, USA; 420301) for 5 min at RT. Tissues were washed in R10, centrifuged, and the pellet was stained with antibodies (Table S1) diluted to 1:100 in PBA [500 mL PBS, 25 mL newborn calf serum (Thermo Fisher Scientific; 26010074), and 1 mL of 10% sodium azide (Sigma-Aldrich, S2002-100G)] for 30 min at 4 °C. Samples were washed with PBA and measured using BD FACSCanto (BD Biosciences, Woburn, MA, USA). Lymphocytes were gated from forward scatter area versus side scatter area. T cells were gated as CD45.2^+^ CD3^+^ cells to access CD4^+^ and CD8^+^ T cell populations.

## 3. Results

### 3.1. TSLP Induction Reduces Tumor Burden in a Mouse Model of Spontaneous Lung Carcinogenesis

**Figure 1:**
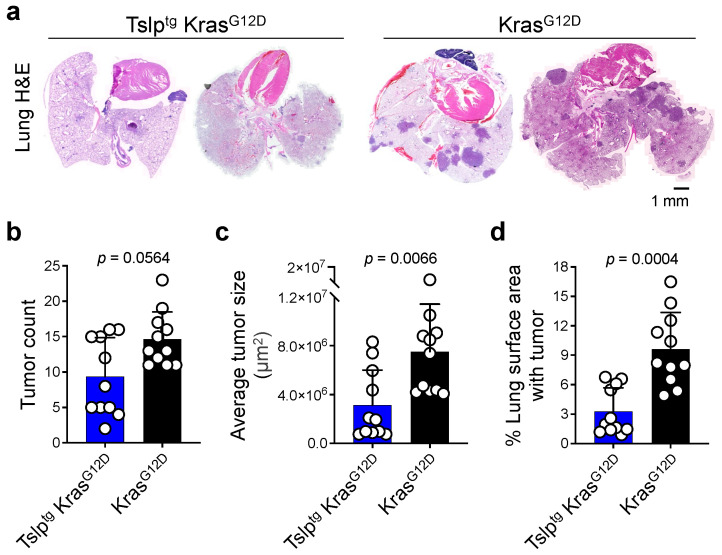
TSLP induction reduces the tumor burden in Kras^G12D^ lungs. (**a**) Representative images of H&E-stained lung of Tslp^tg^ Kras^G12D^ and Kras^G12D^ mice (scale bar: 1 mm). (**b**) Number of tumors in Tslp^tg^ Kras^G12D^ (*n* = 11) and Kras^G12D^ (*n* = 11) lungs. (**c**) Average size of tumors in Tslp^tg^ Kras^G12D^ (*n* = 11) and Kras^G12D^ (*n* = 11) lungs. (**d**) Percentage of the lung surface area with tumor in Tslp^tg^ Kras^G12D^ (*n* = 11) and Kras^G12D^ (*n* = 11) mice. Bar graphs show mean + SD, Mann Whitney *U* test.

To study the impact of systemic TSLP induction on early lung carcinogenesis, we used Kras^G12D^ mice, which develop a spontaneous mutation at position 12 of the Kristen Rat Sarcoma oncogene [22]. This somatic activation of Kras oncogene recapitulates spontaneous oncogene activation in humans [22]. We crossed Kras^G12D^ mice with Tslp^tg^ mice that overexpress TSLP in the skin leading to high circulating TSLP levels [10]. Kras^G12D^ mice develop atypical alveolar hyperplasia as early as two weeks after birth and these lesions progress to adenocarcinomas by postnatal day thirty [22]. Tslp^tg^ Kras^G12D^ (test) and Kras^G12D^ (control) mice were harvested at postnatal day 100, and average lung tumor size, tumor counts, and tumor surface area as a percentage of total lung surface area were recorded. Tslp^tg^ Kras^G12D^ lungs showed a marked reduction in tumor burden with better-preserved lung architecture compared with Kras^G12D^ lungs (Figure 1a). Although TSLP’s effect on suppressing tumor initiation in the lung was marginal (reflected in the number of tumor foci developed in the lung), we found TSLP induction to strongly suppress lung tumor promotion reflected in average tumor size and percentage of the lung surface area covered by tumors (Figure 1b–d). Therefore, systemic TSLP induction substantially suppressed Kras-driven lung tumor promotion. 

### 3.2. TSLP Induction Arrests Tumor Development at an Early Adenoma-like Stage 

Spontaneous activation of an oncogenic allele of Kras results in early-onset lung cancer in Kras^G12D^ mice, with tumors harboring similar histopathological features as human non-small cell lung cancer (NSCLC) [22]. These mice develop hyperplastic lesions in the alveolar epithelium composed of mild dysplasia without disruption of the surrounding lung architecture and closely resemble atypical alveolar hyperplasia (AAH), which have been suggested to be precursors of lung adenocarcinoma in humans [23,24]. AAH progresses to small tumors termed alveolar adenoma, with more densely packed atypical cells and smaller gaps between the basement membrane of type II pneumocytes [22]. Some alveolar adenomas develop into larger, less differentiated tumors, referred to as adenocarcinomas [22]. The slow growth kinetics and human-like morphological progression of the lung tumors in Kras^G12D^ mice provided a suitable model to investigate the mechanism by which TSLP suppressed the early stages of lung carcinogenesis.

**Figure 2:**
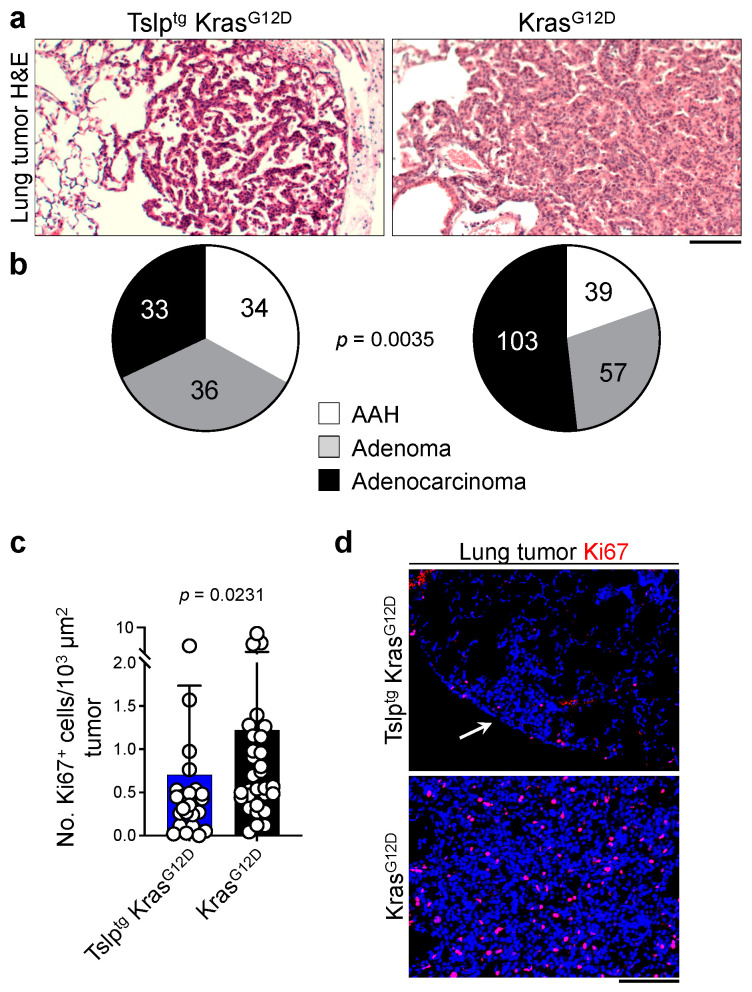
TSLP induction blocks lung cancer progression, which is associated with a decrease in tumor cell proliferation. (**a**) Representative images of H&E-stained tumors of Tslp^tg^ Kras^G12D^ and Kras^G12D^ mice (scale bar: 100 µm). (**b**) Histological grade distribution of Tslp^tg^ Kras^G12D^ (*n* = 103) and Kras^G12D^ (*n* = 199) lung tumors (Fisher’s exact test). The number of tumors in each histological grade is listed on the pie charts. (**c**) The number of Ki67^+^ cells in Tslp^tg^ Kras^G12D^ (*n* = 26) and Kras^G12D^ (*n* = 30) tumors per 10^3^ µm^2^ tumor surface area (Bar graph shows mean + SD, Mann Whitney *U* test). (**d**) Representative images of Ki67-stained tumors in Tslp^tg^ Kras^G12D^ and Kras^G12D^ lungs. Arrow points to atypical alveolar hyperplasia (AAH) in Tslp^tg^ Kras^G12D^ lung. DAPI (blue) stains the cell nuclei, scale bar: 100 µm.

To determine the effects of TSLP on the histopathological progression of lung tumors, we utilized a grading system based on the defined Kras^G12D^ tumor progression in the lung (Figure S1). Interestingly, we observed that the majority of the lung tumors in Tslp^tg^ Kras^G12D^ mice were of AAH and adenoma types while the majority of lung tumors in Kras^G12D^ mice were high-grade adenocarcinoma (Figure 2a,b). To further examine the mechanism of TSLP-mediated cancer suppression in the lung, we stained the Tslp^tg^ Kras^G12D^ and Kras^G12D^ lungs with TUNEL (a marker for cell death) and Ki67 (a marker for cell proliferation). Although we did not detect any increase in tumor cell death in Tslp^tg^ Kras^G12D^ lungs (data not shown), we detected a significant reduction in tumor cell proliferation in Tslp^tg^ Kras^G12D^ compared with Kras^G12D^ lungs (Figure 2c,d). These results suggest that TSLP induction leads to lung cancer suppression by blocking tumor cell proliferation and cancer progression instead of cytotoxicity.

### 3.3. Tslp^tg^ Kras^G12D^ Lungs and Tumors Are Highly Infiltrated by CD4^+^ T Cells

**Figure 3:**
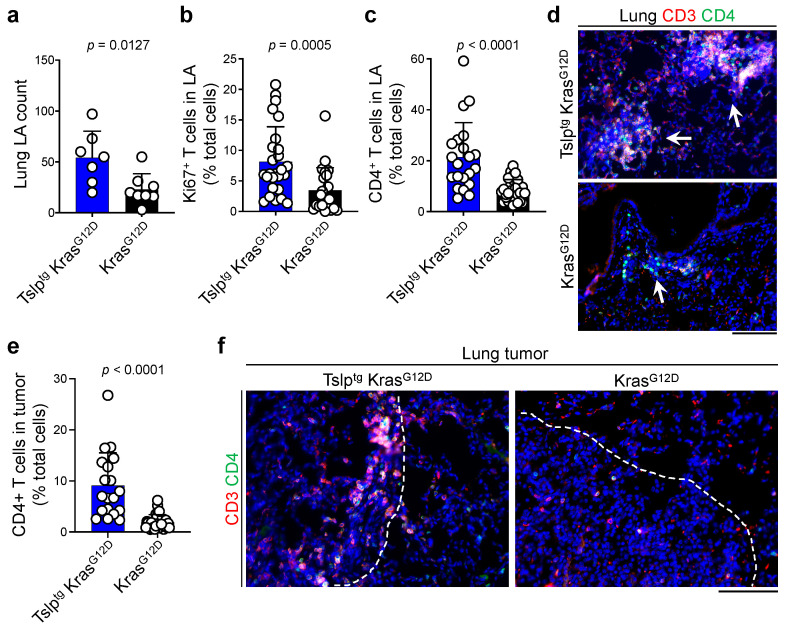
Tslp^tg^ Kras^G12D^ lungs and tumors are highly infiltrated by CD4^+^ T cells. (**a**) The number of lymphoid aggregates (LA) in Tslp^tg^ Kras^G12D^ (*n* = 7) and Kras^G12D^ (*n* = 8) lungs. (**b**) Percentage of Ki67^+^ T cells out of total cells in Tslp^tg^ Kras^G12D^ (*n* = 26) and Kras^G12D^ (*n* = 23) lung lymphoid aggregates. (**c**) Percentage of CD4^+^ T cells out of total cells in Tslp^tg^ Kras^G12D^ (*n* = 21) and Kras^G12D^ (*n* = 29) lung lymphoid aggregates. (**d**) Representative images of CD3 (red) and CD4 (green) stained lymphoid aggregates in Tslp^tg^ Kras^G12D^ and Kras^G12D^ lungs. Arrows point to the lung lymphoid aggregates. Note the larger sizes of lymphoid aggregates in Tslp^tg^ Kras^G12D^ mice. DAPI (blue) stains the cell nuclei, scale bar: 100 µm. (**e**) Percentage of CD4^+^ T cells out of total cells in Tslp^tg^ Kras^G12D^ (*n* = 19) and Kras^G12D^ (*n* = 28) lung tumors. (**f**) Representative images of CD3 (red) and CD4 (green) stained tumors in Tslp^tg^ Kras^G12D^ and Kras^G12D^ lungs. Dashed lines highlight the boundaries of the lung tumors. DAPI (blue) stains the cell nuclei, scale bar: 100 µm. Bar graphs show mean + SD, Mann Whitney *U* test.

Based on our previous findings on the critical role of CD4^+^ T cells in mediating the antitumor effects of TSLP in the skin and breast [9,10], we examined the status of T cell infiltration into the lung and tumors of Tslp^tg^ Kras^G12D^ versus Kras^G12D^ mice. Although Kras^G12D^ lungs showed a trend toward increased number of lymphoid aggregates compared with WT lungs (Figure S2), Tslp^tg^ Kras^G12D^ lungs contained a significantly higher number of T cell-rich lymphoid aggregates compared with Kras^G12D^ lungs (Figure 3a). Notably, Tslp^tg^ lungs had a similar number of lymphoid aggregates compared with WT lungs (Figure S2). In addition, lymphoid aggregates in Tslp^tg^ Kras^G12D^ lungs contained significantly higher T cell proliferation, a higher percentage of CD4^+^ T cells, and were larger compared with lymphoid aggregates in Kras^G12D^ lungs (Figure 3b–d). Consistent with the increased lymphoid aggregate formation in Tslp^tg^ Kras^G12D^ lungs, Tslp^tg^ Kras^G12D^ tumors contained CD4^+^ T cell-rich lymphocytic infiltrates (Figure 3e,f). These results suggest a critical role for TSLP-induced CD4^+^ T cell immunity in blocking lung cancer development.

### 3.4. TSLP-Activated CD4^+^ T Cells Are Required for Suppressing Lung Carcinogenesis

**Figure 4:**
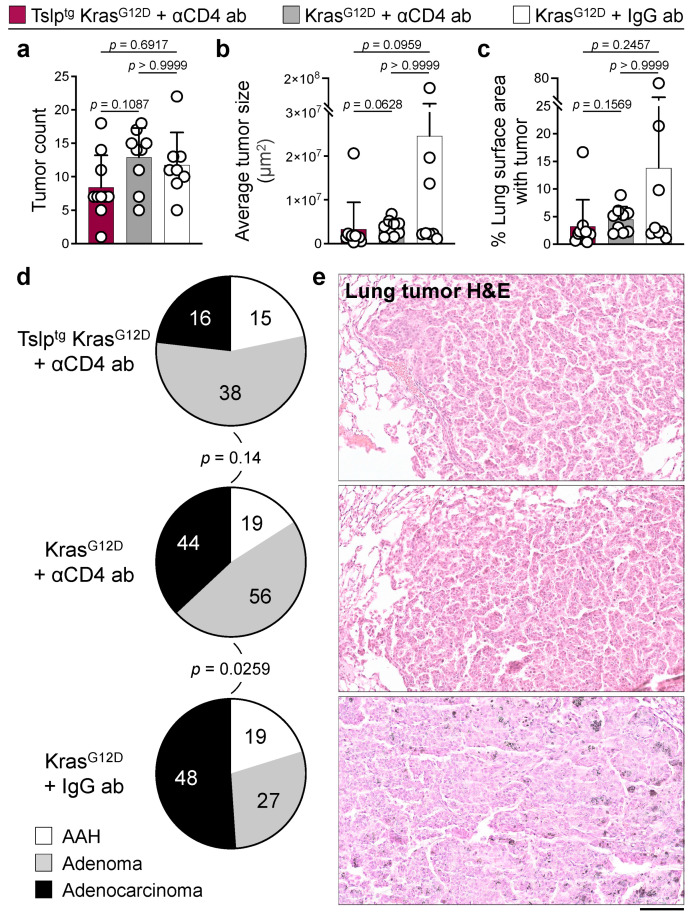
CD4^+^ T cell depletion leads to lung cancer progression in Tslp^tg^ Kras^G12D^ mice. (**a**) Number of tumors in Tslp^tg^ Kras^G12D^ + αCD4 antibody (*n* = 10), Kras^G12D^ + αCD4 antibody (*n* = 9) and Kras^G12D^ + IgG antibody (*n* = 8) lungs. (**b**) Average size of tumors in Tslp^tg^ Kras^G12D^ + αCD4 antibody (*n* = 10), Kras^G12D^ + αCD4 antibody (*n* = 9) and Kras^G12D^ + IgG antibody (*n* = 8) lungs. (**c**) Percentage of the lung surface area with tumor in Tslp^tg^ Kras^G12D^ + αCD4 antibody (*n* = 10), Kras^G12D^ + αCD4 antibody (*n* = 9) and Kras^G12D^ + IgG antibody (*n* = 8) mice. (**d**) Histological grade distribution of Tslp^tg^ Kras^G12D^ + αCD4 antibody (*n* = 69), Kras^G12D^ + αCD4 antibody (*n* = 119) and Kras^G12D^ + IgG antibody (*n* = 94) lung tumors (Fisher’s exact test). The number of tumors in each histological grade is listed on the pie charts. (**e**) Representative images of H&E-stained lung tumors of Tslp^tg^ Kras^G12D^ + αCD4 antibody, Kras^G12D^ + αCD4 antibody and Kras^G12D^ + IgG antibody-treated mice (scale bar: 100 µm). AAH: atypical alveolar hyperplasia, bar graphs show mean + SD, Kruskal-Wallis test with Dunn’s multiple comparison post-hoc test.

To determine whether CD4^+^ T cells were required for the decrease in lung tumor burden observed in Tslp^tg^ Kras^G12D^ mice, we depleted CD4^+^ T cells in Tslp^tg^ Kras^G12D^ and Kras^G12D^ mice. Although long-term αCD4 antibody treatment failed to completely deplete CD4^+^ T cells in mice (Figure S3), the marked reduction in CD4^+^ T cells in Tslp^tg^ Kras^G12D^ mice erased any differences between tumor size and percentage of lung surface area with tumor in Tslp^tg^ Kras^G12D^ compared with Kras^G12D^ mice (Figure 4a–c). Interestingly, CD4^+^ T cell depletion resulted in similar lung tumor grades in Tslp^tg^ Kras^G12D^ compared with Kras^G12D^ mice (Figure 4d,e). Importantly, we observed that, in the absence of TSLP induction, Kras^G12D^ mice treated with αCD4^+^ T cell antibody developed lower grade lung tumors compared with Kras^G12D^ mice treated with IgG control (Figure 4d,e). These results demonstrate that TSLP-activated CD4^+^ T cells play an essential role in blocking lung cancer progression in Tslp^tg^ Kras^G12D^ mice. However, baseline CD4^+^ T cells present in Kras^G12D^ lungs may have a tumor-promoting function [16,25,26]. 

## 4. Discussion

Immunomodulatory agents that can block early stages of cancer development provide a novel strategy to enable cancer immunoprevention. Due to reduced antigen presentation and low co-stimulatory molecule expression, lung cancer cells evade cytotoxic immunity [27]. *KRAS^G12D^* and *TP53* co-mutation are associated with reduced immune cell infiltrates in the human lung adenocarcinoma [28]. In addition, patients with lung adenocarcinoma lack antitumor innate immunity at baseline including tumor-infiltrating NK cells, which are less cytolytic due to low expression of granzyme B and interferon-gamma [29]. In this report, we have shown that TSLP cytokine induction can mount a strong antitumor immunity in an oncogene-driven in vivo model of spontaneous lung adenocarcinoma. Our findings demonstrate that cytokine induction, in the context of oncogene-driven early carcinogenesis, can lead to T cell infiltration into the tumor and the suppression of cancer progression from premalignancy to invasive disease. This is also significant because current cancer immunotherapies for lung cancer are not effective against early disease due to their poor immune infiltration at baseline. Our results indicate that cytokines can trigger a potent antitumor adaptive immune response during the early stages of lung cancer development. When delivered between the time of cancer diagnosis and surgical resection, the cytokine inductive treatment may provide an effective strategy for treating the malignant lesion as well as preventing its recurrence. 

We find that TSLP induction not only suppresses lung tumor initiation as demonstrated by lower tumor counts in mice expressing the *Tslp* transgene but also results in the development of smaller, more differentiated tumors. This is of particular relevance because lower histological grades in lung cancers are associated with lower recurrence rates [30] and better survival outcomes [31]. We also show higher CD4^+^ T cell infiltrates in lung tumors and lymphoid aggregate formation in the lungs of Tslp^tg^ Kras^G12D^ compared with Kras^G12D^ mice that do not overexpress TSLP. This is consistent with TSLP’s role in the induction of Th2 cell proliferation and activation in barrier organs [14]. As a cardinal cytokine in the pathogenesis of allergic diseases, the cancer-protective effect of TSLP in the lung provides an explanation for the epidemiological observation of an inverse relationship between allergic disease and lung cancer risk [12,13]. Importantly, the significantly higher lymphoid aggregate counts in Tslp^tg^ Kras^G12D^ compared with Tslp^tg^ mice suggests that TSLP-activated T cells in the lungs are responding to the tumor antigens as opposed to a nonspecific allergic inflammation caused by TSLP overexpression alone. Our results put forward TSLP induction as a potential therapeutic approach that can trigger a lasting antitumor immune response capable of bringing the adaptive immune cells to recognize and suppress early stages of lung carcinogenesis. Considering a significant reduction in tumor cell proliferation in Tslp^tg^ Kras^G12D^ tumors compared with Kras^G12D^ tumors, cytotoxicity is unlikely to be the main mode of antitumor immunity downstream of TSLP induction in the lung. Future studies are warranted to investigate the precise nature of antitumor CD4^+^ T cell immunity induced by TSLP and determine the impact of TSLP on other immune cells including CD8^+^ T cells in suppressing lung carcinogenesis.

It is important to note the emerging role of CD4^+^ T cells in antitumor immunity, and in particular, their functional versatility in the context of the tumor immune microenvironment. CD4^+^ T cells have been shown to initiate a strong antitumor response in tumors by enhancing the clonal expansion of cytotoxic T lymphocytes as well as directly through the secretion of TNF-α and other tumor-suppressive factors [32]. Additionally, CD4^+^ T cells have been shown to promote the differentiation of effector CD8^+^ T cells in a mouse melanoma model known to be immunogenically cold and poorly responsive to immune checkpoint blockade [33,34]. CD4^+^ T cells are found to be required for immune checkpoint blockade response in the context of a poorly immunogenic T3 sarcoma cell line engineered to express MHCII antigens [35]. Of note, CD4^+^ T cells have been demonstrated to have anti-cancer functions outside their role in stimulating cytotoxic immune cells in cancer. TGF-β blockade leads to CD4^+^ Th2 cell-induced cancer cell hypoxia through the remodeling of the tissue vasculature in an MMTV-PyMt murine breast cancer model [36]. Clinically, the presence of CD4^+^ T cells in the peripheral blood of lung cancer patients before immune checkpoint blockade therapy is associated with increased tumor-infiltrating CD8^+^ T cells and better response to PD-1 therapy [37,38]. We provide evidence to support the critical role of CD4^+^ T cells in antitumor immunity and highlight the need for future investigation into the role of type 2 immunity in the context of early carcinogenesis. We also find induction of broad T cell immunity by TSLP in the lung including CD8^+^ T cells with known antitumor effects. Future studies are warranted to address the role of TSLP-stimulated CD8^+^ T cells in lung carcinogenesis. In addition, understanding the role of TSLP signaling to innate immune cells and Kras mutant tumor cells in regulating lung cancer development is an important area for future investigation. Furthermore, we aim to study the effects of TSLP receptor expression in lung tumor cells, lymphocytes and other antigen presenting cells to understand the role of TSLP signaling to these cell types in the lung tumor microenvironment.

## 5. Conclusions

In conclusion, we have shown that TSLP induction reduces tumor burden and blocks cancer progression in a mouse model of spontaneous lung adenocarcinoma. TSLP induction leads to a massive T cell infiltration into and lymphoid aggregate formation in the tumor-bearing lungs. Finally, we have demonstrated that TSLP-activated CD4^+^ T cells are required to suppress the progression of Kras-driven lung tumors to invasive adenocarcinoma in mice.

## Data Availability

All data needed to evaluate the conclusions in the paper are present in the main text and/or the Supplementary Materials.

## Supplementary Materials

The following are available online at https://www.mdpi.com/article/10.3390/cancers14092173/s1, Figure S1: Representative images of lung tumor histological grades, Figure S2: Lymphoid aggregate numbers in mice lacking Kras^G12D^ oncogene, Figure S3: CD4^+^ T cell depletion platform and the efficacy of long-term CD4^+^ T cell depletion in mice, Table S1: Antibodies used in the study.
