# Supplementary material for: Thymic Stromal Lymphopoietin Induction Suppresses Lung Cancer Development
Source: Cancers (Basel). 2022 Apr 27;14(9):2173. doi: 10.3390/cancers14092173 (PMC9104311; doi:10.3390/cancers14092173)
Supplement: Supplementary file 1 [file cancers-14-02173-s001.zip › cancers-1633435-supplementary.pdf]

cancers

Supplementary Material: Thymic stromal lymphopoietin induction suppresses lung cancer development

Ranya Guennoun, Jennet Hojanazarova, Kathryn E. Trerice, Marjan Azin, Matthew T. McGoldrick, Erik B. Schiferle, Michael P. Stover and Shadmehr Demehri

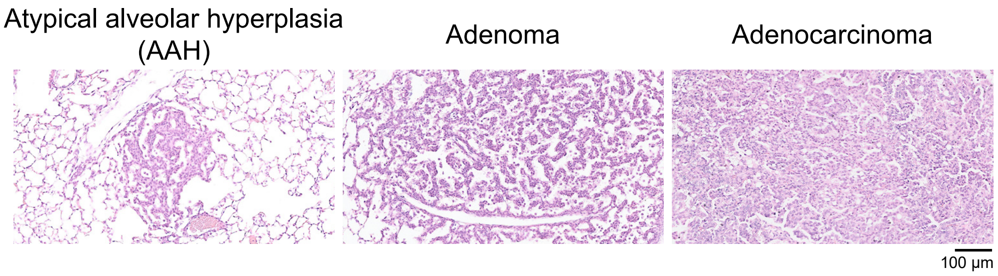

Figure S1. Representative images of lung tumor histological grades.

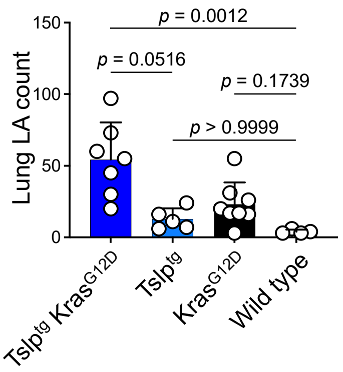

Figure S2. Lymphoid aggregate numbers in mice lacking Kras^{G12D} oncogene. Number of lymphoid aggregates (LA) in Tslp^{tg} Kras^{G12D} (n = 7), Tslp^{tg} (n = 5), Kras^{G12D} (n = 8) and wild-type (n = 4) lungs. Note that Tslp^{tg} Kras^{G12D} and Kras^{G12D} LA counts are also shown in Figure 3a, bar graphs show mean + SD, Kruskal-Wallis test with Dunn's multiple comparison post-hoc test.

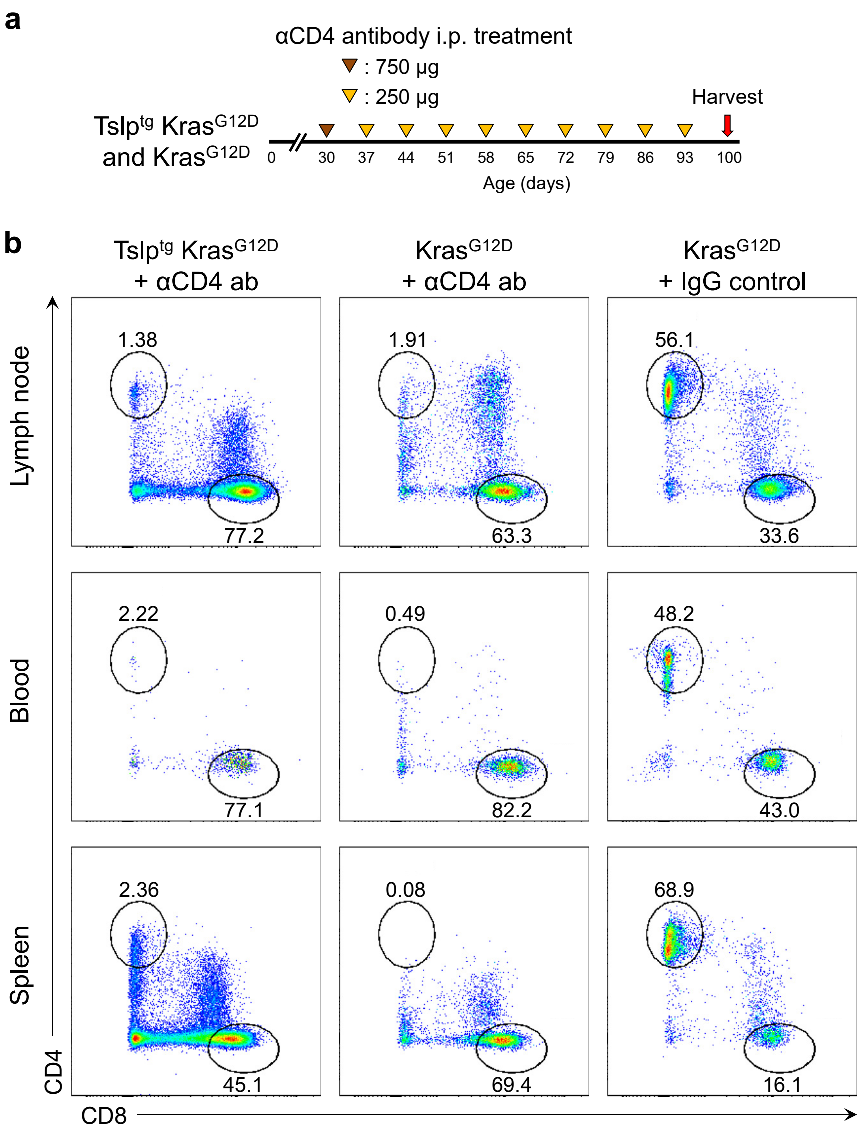

Figure S3. $CD4^+$ T cell depletion platform and the efficacy of long-term $CD4^+$ T cell depletion in mice. (**a**) Schematic diagram of $CD4^+$ T cell depletion in $Tslp^{tg}$ $Kras^{G12D}$ and $Kras^{G12D}$ mice using αCD4 antibody (or IgG control antibody) intraperitoneal injections. (**b**) Representative flow cytometry for T cells in blood, spleen and lymph node of αCD4 antibody-treated $Tslp^{tg}$ $Kras^{G12D}$ and $Kras^{G12D}$ mice compared with IgG control-treated $Kras^{G12D}$ mice at postnatal day 100. Note the partial depletion of $CD4^+$ T cells in αCD4 antibody-treated mice. Numbers on the flow plots show the percentage of the cells within each gate.

Table S1. Antibodies used in the study.

Flow cytometry Antibodies	Conjugate	Clone	Manufacturer	Cat #	Isotype	RRID
CD3	AF700	17A2	BioLegend	100216	Rat IgG2b, k	AB_493697
CD4	APC-Cy7	RM4-5	BioLegend	100526	Rat IgG2a, k	AB_312727
CD8	FITC	53-6.7	BioLegend	100706	Rat IgG2a, k	AB_312745
CD45.2	eF450	104	Thermo Fisher	48-0454-82	Mouse IgG2a, k	AB_11042125
**Immunofluorescence Antibodies**						
CD3	Purified	CD3-12	Abcam, Waltham, MA, USA	Ab11089	Rat IgG1	AB_369097
CD4	Purified	EPR19514	Abcam	Ab183685	Rabbit IgG	AB_2686917
Ki67	Purified	Polyclonal	Abcam	Ab15580	Rabbit IgG	AB_443209
**Depleting Antibodies**						
αCD4 antibody	-	GK1.5	BioXCell, Lebanon, NH, USA	BE0003-1	Rat IgG2b,k	AB_110763
